# Supplementary material for: Development of a Polygenic Risk Score for BMI to Assess the Genetic Susceptibility to Obesity and Related Diseases in the Korean Population
Source: Int J Mol Sci. 2023 Jul 17;24(14):11560. doi: 10.3390/ijms241411560 (PMC10380444; doi:10.3390/ijms241411560)
Supplement: Supplementary file 1 [file ijms-24-11560-s001.zip › Supplementary Figure S1.pptx]

## Slide 1
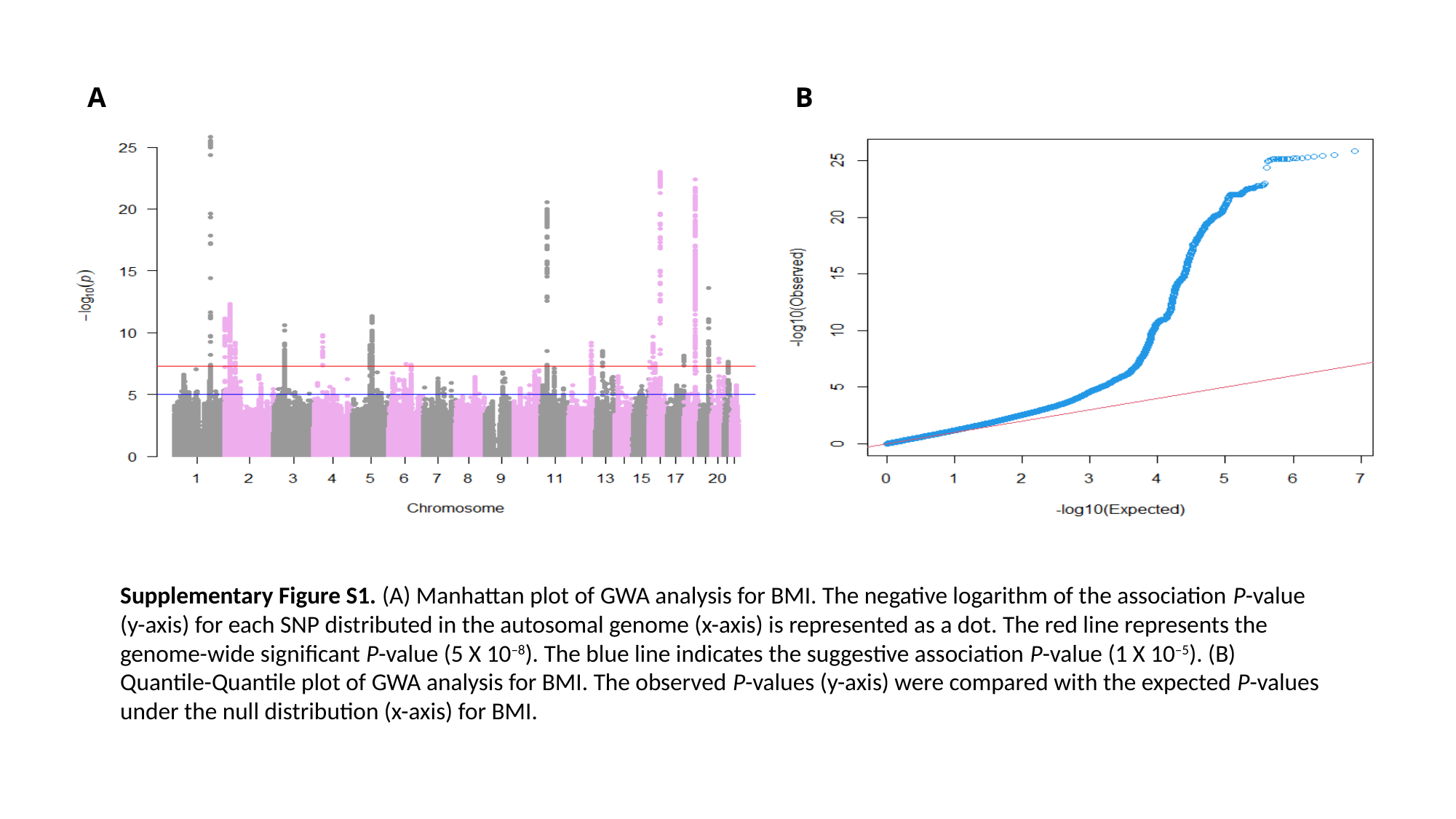

A
B
Supplementary Figure S1. (A) Manhattan plot of GWA analysis for BMI. The negative logarithm of the association P-value (y-axis) for each SNP distributed in the autosomal genome (x-axis) is represented as a dot. The red line represents the genome-wide significant P-value (5 X 10–8). The blue line indicates the suggestive association P-value (1 X 10–5). (B) Quantile-Quantile plot of GWA analysis for BMI. The observed P-values (y-axis) were compared with the expected P-values under the null distribution (x-axis) for BMI.
