# Supplementary material for: Development of a Polygenic Risk Score for BMI to Assess the Genetic Susceptibility to Obesity and Related Diseases in the Korean Population
Source: Int J Mol Sci. 2023 Jul 17;24(14):11560. doi: 10.3390/ijms241411560 (PMC10380444; doi:10.3390/ijms241411560)
Supplement: Supplementary file 1 [file ijms-24-11560-s001.zip › Supplementary Figure S2.pptx.pptx]

## Slide 1
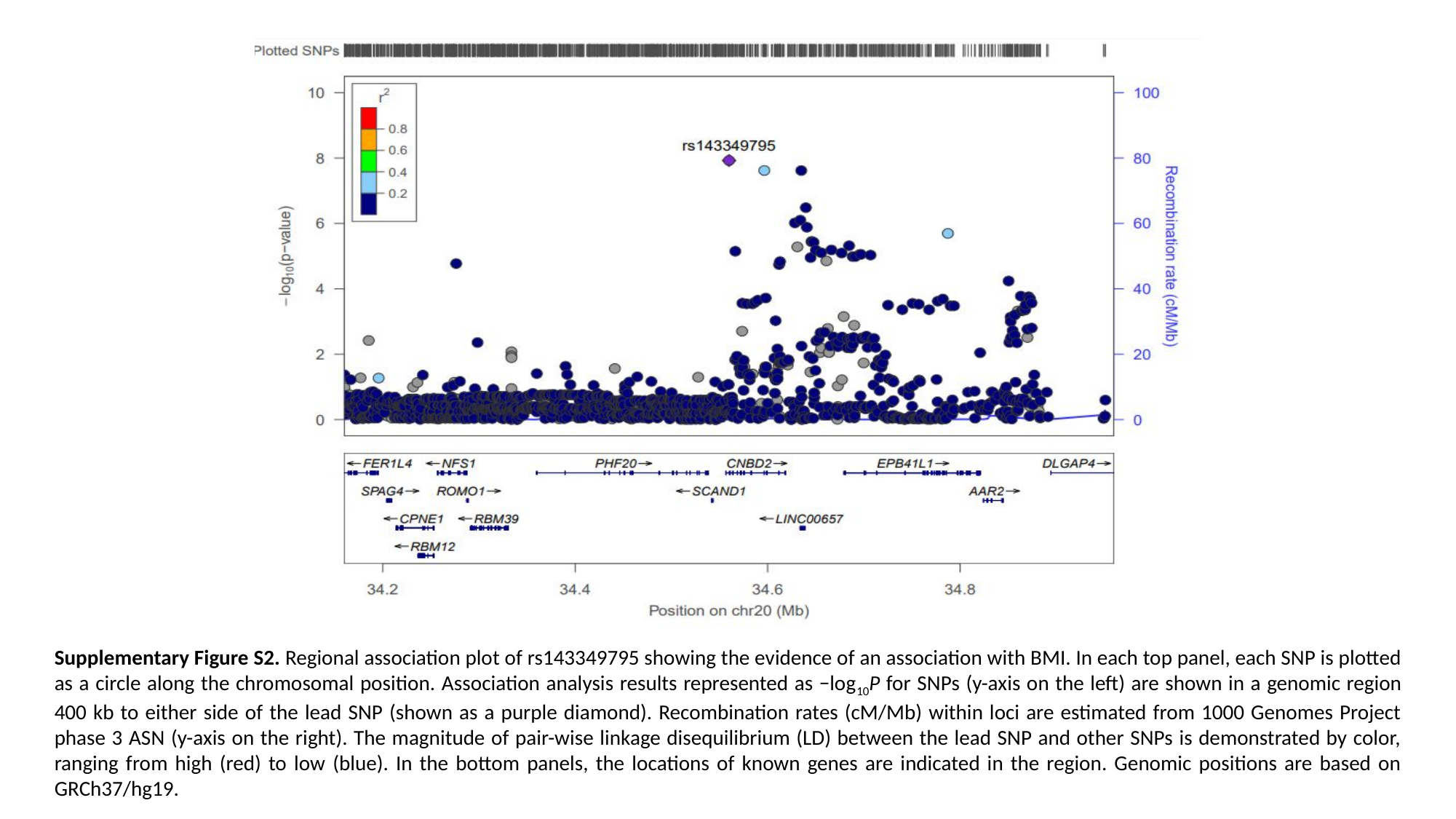

Supplementary Figure S2. Regional association plot of rs143349795 showing the evidence of an association with BMI. In each top panel, each SNP is plotted as a circle along the chromosomal position. Association analysis results represented as −log10P for SNPs (y-axis on the left) are shown in a genomic region 400 kb to either side of the lead SNP (shown as a purple diamond). Recombination rates (cM/Mb) within loci are estimated from 1000 Genomes Project phase 3 ASN (y-axis on the right). The magnitude of pair-wise linkage disequilibrium (LD) between the lead SNP and other SNPs is demonstrated by color, ranging from high (red) to low (blue). In the bottom panels, the locations of known genes are indicated in the region. Genomic positions are based on GRCh37/hg19.
