# Supplementary material for: Development of a Polygenic Risk Score for BMI to Assess the Genetic Susceptibility to Obesity and Related Diseases in the Korean Population
Source: Int J Mol Sci. 2023 Jul 17;24(14):11560. doi: 10.3390/ijms241411560 (PMC10380444; doi:10.3390/ijms241411560)
Supplement: Supplementary file 1 [file ijms-24-11560-s001.zip › Supplementary Table S1.pptx.pdf]

Supplementary Table S1. Results of association analysis between SNP and BMI adjusting for sex, age, and recruitment area using KBA dataset of 57,110 individuals from HEXA cohort. A total of 20 lead SNPs showing genome-wide significant association were selected from about 8M SNPs used for association analysis.

CHR	SNP	BP	RA	MAF	Beta	SE	*P*	Candidate Gene
1	rs633715	177852580	C	0.147	0.207	0.019	1.40×10^{-26}	*SEC16B*
16	rs55872725	53809123	T	0.166	0.255	0.025	9.79×10^{-24}	*FTO*
18	rs476828	57852587	C	0.205	0.188	0.019	3.87×10^{-23}	*MC4R*
11	rs6265	27679916	C	0.488	0.160	0.017	2.67×10^{-21}	*BDNF-AS - BDNF*
19	rs35560038	46175046	A	0.468	0.130	0.017	2.55×10^{-14}	*GIPR*
2	rs713586	25158008	C	0.478	0.122	0.017	4.48×10^{-13}	*ADCY3 - DNAJC27*
5	rs261967	95850250	C	0.465	0.117	0.017	4.32×10^{-12}	*PCSK1 - CAST*
2	rs7570198	648198	T	0.087	0.200	0.029	7.41×10^{-12}	*LOC105373352 - TMEM18*
3	rs3755804	52866289	T	0.217	0.124	0.019	2.46×10^{-11}	*ITIH4 - MUSTN1*
4	rs10938397	45182527	G	0.303	0.120	0.019	1.57×10^{-10}	*PRDX4P1 - THAP12P9*
16	rs13329725	20257273	A	0.191	0.135	0.021	2.18×10^{-10}	*SNRPEP3*
12	rs11066132	112468206	C	0.172	0.144	0.023	6.07×10^{-10}	*NAA25*
2	rs7560575	54142030	T	0.012	0.496	0.080	6.60×10^{-10}	*PSME4*
5	rs112862634	87970352	C	0.474	0.103	0.017	1.05×10^{-9}	*LINC00461*
13	rs4883723	54084032	A	0.282	0.110	0.019	3.44×10^{-9}	*ZNF646P1*
17	rs35867081	79047278	A	0.298	0.101	0.018	7.31×10^{-9}	*BAIAP2*
20	rs143349795	34559849	A	0.012	0.309	0.054	1.19×10^{-8}	*CNBD2*
16	rs2540034	4022694	T	0.313	0.098	0.018	2.21×10^{-8}	*ADCY9*
21	rs2211691	35609354	T	0.485	0.094	0.017	2.30×10^{-8}	*LINC00310*
6	rs4946936	109003321	C	0.284	0.111	0.020	4.00×10^{-8}	*FOXO3*

Results of association analysis are limited to SNPs with P-value $< 5.0 \times 10^{-8}$. The SNP ID and chromosomal position (BP) are based on NCBI genome build 37/hg19. Abbreviations are as follows: CHR, chromosome; SNP, single nucleotide polymorphism; BP, physical position (base-pair); RA, risk allele; MAF, minor allele frequency; SE, standard error.
